# Phytochemical Compounds of Raw Versus Methanol-Extracted Kelulut, Tualang, and Manuka Honeys

**DOI:** 10.7759/cureus.38297

**Published:** 2023-04-29

**Authors:** Liu Imm Chu, Zurairah Berahim, Suharni Mohamad, Wan Nazatul Shima Shahidan, Mohd Firdaus Yhaya, Siti Lailatul Akmar Zainuddin

**Affiliations:** 1 School of Dental Sciences, Universiti Sains Malaysia, Kubang Kerian, MYS

**Keywords:** phytochemical compounds, extracted honey, manuka honey, tualang honey, kelulut honey

## Abstract

Honey has been widely used for medicinal purposes since ancient times. It is produced by stinging bees or stingless bees by processing the collected nectar or plant sap in their bodies into raw honey. Extraction of honey will result in the pooling of crude volatile bioactive materials that could enhance its benefits. This work aims to compare the phytochemical characteristics of raw and methanol-extracted honeys in the Kelulut, Tualang and Manuka honeys. All types of raw honey samples were extracted by using the methanol extraction method and both groups were analysed using gas chromatography/mass spectrometry (GC/MS) at the National Poison Centre, Universiti Sains Malaysia, Malaysia. The findings showed that 23 compounds were identified in raw Kelulut honey and 18 compounds in methanol-extracted Kelulut honey; 28 compounds were identified in raw Tualang honey and 29 compounds in methanol-extracted Tualang honey; 19 compounds in raw Manuka honey and 22 compounds in methanol-extracted Manuka honey. There were differences in the phytochemical substances detected in raw and methanol-extracted honeys. The major compounds in raw honeys were mostly from the ketone, alcohol, and ester groups, whereas the ketone group was dominant in methanol-extracted honeys. Most bioactive substances identified in the methanol-extracted variant of honeys were more concentrated than the raw variant. A majority of these substances have antimicrobial characteristics.

## Introduction

Bees collect sweet substances or saps from flower nectar, plants, and other materials and convert them into honey which is valuable for mankind. Honey constituents differ and are highly dependent on the plants bees use for feeding [1]. Sugar is a major constituent of honey in addition to other substances like amino acids, vitamins, phenolic acids, enzymes, minerals, and flavonoids. Honey has a high phenolic content and significant antioxidant properties [2] as well as effectiveness against bacteria, fungi, and cancer [3-6].

Honeybees can be divided into stinging bees and stingless groups. The sting bees belong to the *Apis* genus and have a stinger on their abdomen, whereas stingless bees do not have a stinger and are classified into *Melipona* and *Trigona*. Both have an important role in flower pollination. In terms of therapeutic quality, stingless bee honey was found to be on par with sting bee honey [7]. Both sting and stingless bee honey exhibit significant antibacterial, antioxidant, anticancer, and antiatherogenic activities, which may be attributed partly to their phenolic content [8].

In Malaysia, Kelulut honey is the local honey produced by domestic stingless bees from the *Trigona* genus. Kelulut honey has a relatively high liquid form and is sour compared to other varieties. On the other hand, Tualang honey is produced by the wild sting bees, *Apis dorsata*, that fabricate hives high within the parts of the Tualang tree. Because the bees forage in the wild, it is believed that their honey contains an abundance of high-quality and beneficial components [9]. Manuka honey is New Zealand honey produced by *Apis mellifera* from the nectar of Manuka flowers. The honey also has other unique features, like an extraordinarily high level of methylglyoxal (MGO) formed from dihydroxyacetone (DHA), which correlates with antibacterial activity [10]. Manuka honey is known as "medical-grade honey" and has been used widely [11].

Raw honey has significant quantities of water and sugar and minute quantities of other bioactive chemicals. Hence, extraction techniques can be used to obtain significant quantities of any bioactive constituent. Similar to other chemical reactions, it is necessary to examine any potential post-process phytochemical alterations. The present study used gas chromatography-mass spectrometry (GC-MS) assessment to validate the phytochemicals in the raw and methanol-extracted honey variants. This information may aid in a better understanding of the properties of honey for future pharmacological applications.

## Materials and methods

Kelulut honey was purchased from Kelulut Apiary, Kem Desa Pahlawan, Kelantan, Malaysia; Tualang honey was purchased from AgroMas, Federal Agriculture and Marketing Authority (FAMA), Malaysia; and Manuka honey (Manuka Health, Auckland, Zealand) was purchased from a local pharmacy. All honeys were obtained around March 2020.

pH measurement

The pH value of raw and methanol-extracted honeys was measured using Hanna pH 211 microprocessor pH meter (Hanna Instruments, Woonsocket, Rhode Island, United States).

Methanol extraction of honey

The extraction technique was based on Mohapatra et al. (2011) [12]. The raw honey was weighed at 10 g in a centrifuge tube. Then, 25 ml of methanol was added to the honey and vortexed until homogeneously mixed. The honey-methanol mixture was subjected to centrifugation at 3000 rpm for 10 minutes at 25°C. The supernatant thereafter was collected and transferred into a new 50 ml centrifuge tube, then subjected to a concentrator for eight hours (repeated every two hours) in order to completely discard the methanol residues. Lastly, it was subjected to overnight lyophilisation.

GC-MS analysis

All raw and methanol-extracted samples were sent for GC-MS analysis at the National Poison Centre, Gelugor, Malaysia. The samples were analysed using a Hewlett Packard 6890 series gas chromatograph with 5973N mass selective detector and ChemStation Data System (Agilent Technologies Inc., Santa Clara, California, United States).

## Results

pH analysis of raw and crude methanolic-extracted honey

All types of honey samples (raw Kelulut honey (RKH), methanol-extracted Kelulut honey (EKH), raw Tualang honey (RTH), methanol-extracted Tualang honey (ETH), raw Manuka honey (RMH), and methanol-extracted Manuka honey (EMH)) in this experiment were acidic, with a pH range of 3.44 to 4.38. In general, raw honeys were found to be more acidic than methanol-extracted honey (Table 1). The RKH and EKH had the lowest pH values, indicating that they were the most acidic.

**Table 1:** pH value of honey.

Types of honey	pH value	Types of honey	pH value
Raw Kelulut honey	3.44	Methanol-extracted Kelulut honey	3.93
Raw Tualang honey	3.58	Methanol-extracted Tualang honey	4.10
Raw Manuka honey	3.66	Methanol-extracted Manuka honey	4.38

Compounds analysis from raw and crude methanolic extract honey

The GC-MS assessment (Table 2) indicated the presence of 23 chemicals in RKH, while the methanol-extracted variant had 18 chemicals. The highest peak area in RKH is 5-(hydroxymethyl)-2-furancarboxaldehyde, which was 13.52%, and in EKH was 29.29%. Most of the bioactive compounds in RKH and EKH are in the ketone group, followed by alcohol and ester groups. Based on the results, the peak area of most compounds in EKH was higher compared to RKH.

**Table 2:** Comparison of peak area (%) between raw and methanol-extracted Kelulut, Tualang and Manuka honey. RKH=raw Kelulut honey, EKH=methanol-extracted Kelulut honey, RMH=raw Manuka honey, EMH=methanol-extracted Manuka honey, RTH=raw Tualang honey, ETH=methanol-extracted Tualang honey.

Functional Group	Compounds	Types of Honey (Percentage of Total %)					
		RKH	EKH	RMH	EMH	RTH	ETH
Ester (methyl ester)	Hexadecanoic acid, methyl ester	0.75	-	4.29	-	0.61	10.46
	9-Octadecenoic acid, methyl ester	-	-	-	-	0.91	2.67
	Octadecanoic acid, methyl ester	-	-	-	-	0.70	-
	9-Octadecenoic acid (Z)-, methyl ester	1.63	-	-	-	-	-
	Dodecanoic acid, methyl ester	-	-	0.85	-	-	-
	Tetradecanoic acid, methyl ester	-	-	0.12	-	-	-
	12-Octadecenoic acid, methyl ester	-	-	-	3.20	-	-
Ester	Butyrolactone	-	-	-	-	0.02	-
	Hexadecanoic acid, ethyl ester	-	-	-	-	-	4.04
	Octanoic acid, 2-ethylhexyl ester	-	-	0.13	-	-	-
	Lauric acid, N-octyl ester	-	-	22.41	-	-	-
	Methacrylic acid, hexadeyl ester	-	-	3.63	-	-	-
	Fumaric acid, 2-ethylhexyl octyl ester	-	-	11.92	-	-	-
	Methacrylic acid, heptadeyl ester	-	-	0.30	-	-	-
	Hexyl ester of 2-ethyl-palmitic acid	-	-	8.35	-	-	-
Ester, Ketone	Propanoic acid, 2-oxo-, methyl ester	3.60	-	-	-	3.73	-
	1-(Acetyloxy)-2-propanone	0.30	0.75	-	-	0.92	1.46
Ester, Fluoroalkane	Eicosyl pentafluoropropionate	-	-	-	0.01	-	-
Ester Furan	5-Formyl-2-furfurylmethanoate	-	-	-	-	-	6.04
Aldehyde	Benzeneacetaldehyde	3.26	0.12	-	-	-	0.45
Ketone	1,2-Cyclohexanedione	1.14	-	-	2.38	0.89	0.81
	3-Octanone	-	-	-	-	-	1.60
	diisopropyl propional	2.87	-	-	-	-	-
Aldehyde, Furan	Furfural	0.39	3.58	-	7.43	0.04	3.89
	2-Furancarboxaldehyde	-	-	-	-	0.55	-
	5 Methyl furfural	-	0.42	-	-	0.42	-
	5-Methyl-2-furancarboxaldehyde	-	-	-	-	-	1.06
Ketone, Furan	1-(2-Furanyl)-ethanone	-	0.16	-	0.25	0.06	0.45
	2-Acetyl furan	-	-	-	-	-	0.74
	(2H)-furan-2-one	-	2.43	-	3.97	-	1.85
	2(5H)-furanone	-	-		0.38	-	0.73
	Protoanemonine	0.35	0.60	-	-	0.64	1.43
Ketone, Pyran	Cyclopenta[c]pyran-3(5H)-one	-	-	-	-	-	0.36
	5,6-Dihydro-2H-pyran-2-one	-	-	-	1.54	-	-
Ketone, Amino	2,4-Cycloheptadien-1-one,2-(diethylamino)-7-methyl-7-phenyl-	-	-	1.86	-	-	-
Alcohol, Ketone	2-Cyclopenten-1-one, 2-hydroxy-3-methyl-	-	-	-	-	1.89	-
	1,3-Dihydroxy-2-propanone	18.15	-	-	6.15	18.46	-
	Acetol	-	2.15	-	-	-	1.85
Alcohol, Ketone Furan,	2,4-Dihydroxy-2,5-dimethyl-3(2H)-furan-3-one	-	0.38	-	1.34	0.47	0.97
	Furaneol	-	-	-	4.29	-	2.81
	Hydroxy dimethyl furanone	-	3.19	-	-	-	-
Alcohol, Ketone, Pyran	4H-Pyran-4-one, 2,3-dihydro-3,5-dihydroxy-6-methyl-	4.69	-	-	-	0.38	-
	2,3-Dihydro-5-hydroxy-6-methyl-4H-pyran-4-one	-	8.37	-	1.82	-	-
Alcohol, Furan, Aldehyde	5-(Hydroxymethyl)-2-furancarboxaldehyde	13.52	29.29	-	44.32	16.15	28.42
Alcohol	1-Hexanol,2-ethyl-	-	-	0.49	-	-	-
	Menthol	-	-	0.79	-	-	-
	1-Hentriacontanol	-	-	-	0.10	-	-
Alcohol, Furan	trans-Linalool oxide	-	-	-	-	0.47	0.40
	2-Furanmethanol	-	-	-	-	-	1.15
Alcohol, Ether	Ethyl.alpha.-d-glucopyranoside	-	-	-	-	5.82	-
	1,3-Dihydroxyacetone dimer	-	7.64	-	-	-	-
Alcohol, Pyran	.beta.-D-Glucopyranose	-	9.76	-	-	-	-
Alcohol, Ester	3-Deoxy-d-mannoic lactone	-	-	-	-	9.32	-
	9-Octadecenoic acid (Z)- ,2,3-dihydroxypropyl ester	0.77	-	-	-	0.47	-
Alcohol, Benzoic acid	2-(5-tert-Butyl-4-hydroxy-2-methylphenyl)benzoic acid	-	-	6.14	-	-	-
Alkane	Tetracontane,3,5,24-trimethyl-	-	-	-	-	0.45	-
	Eicosane	0.77	-	-	-	0.14	0.15
	Tetratetracontane	-	-	-	-	0.08	-
	Docosane, 11-butyl-	-	-	-	-	0.02	-
	Nonadecane	-	-	-	-	-	0.82
	1-Docosane	-	-	-	0.09	-	0.96
	Cyclododecane	2.08	-	-	-	-	-
	Tetradecane	0.45	-	-	-	-	-
	Dodecane	-	-	0.86	-	-	-
	Tridecane	-	-	1.60	-	-	-
	Hexadecane,7,9-dimethyl-	-	-	0.36	-	-	-
	Octadecane	-	-	-	0.02	-	-
Alkene	7-Hexadecene, (Z)-	5.66	-	-	-	-	-
	1,19-Eicosadiene	-	0.23	-	-	-	-
Carboxylic acid	cis-Vaccenic acid	-	-	-	-	5.02	-
	Formic acid	-	-	-	-	-	1.63
	Acetic acid	-	2.49	-	2.53	-	2.80
	9-Octadecenoic acid (Z)-	-	-	-	-	-	4.49
	Palmitic acid	2.56	-	-	-	-	-
	9-Octadecenoic acid, (E)-	0.61	-	-	-	-	-
	Stearic acid	3.66	-	-	-	-	-
	Hexadecanoic acid	-	4.11	-	-	-	-
	Oleic acid	-	-	8.96	-	-	-
	n-Hexadecanoic acid	-	-	-	0.65	-	-
Carboxylic acid, Ether	Butanoic acid, 4-butoxy-	10.48	-	-	-	-	-
Carboxylic acid, Furan	3-Furancarboxylic acid	-	-	-	0.67	-	-
	3-Methyl-2-furoic acid	-	-	-	11.90	-	-
Phenol	Pyrocatechol	-	-	-	-	2.96	-
Phenol, Methoxy	Butylated hydroxyanisole	0.58	-	-	-	-	-
Phenol, Ether	Vitamin E	0.32	0.05	-	0.05	0.12	0.30
Phenol, Amines, Ether	Benzoic acid, 4-hydroxy-3,5-dimethoxy-, hydrazide	-	-	-	0.76	-	-
Amide, Sulfide, Chloroalkene	5-Chloro-2-methyl-3(2H)-isothiazolone	-	-	1.92	-	-	-
Nitrile	2,3-Diethyl-2,3-dimethylsuccinonitrile	-	-	0.61	-	-	-

Twenty-eight compounds were detected in RTH while 29 compounds were found in ETH; 5-(hydroxymethyl)-2-furancarboxaldehyde was recorded as the highest peak area in ETH, and 28.42% and 16.15% in RTH. Most of the compounds in RTH are in alcohol groups, followed by ketone and ester, while most of the compounds in ETH are in the ketone group, followed by alcohol and ester groups. Based on the results, the peak area of most compounds in ETH was higher compared to RTH.

The identified compounds found in RMH were 19 compounds and in EMH were 22 compounds. The methanol-extracted honey contains more volatile compounds compared to raw honey. Interestingly, results showed that the compounds presented in RMH and EMH were totally different. The major compound in RMH was lauric acid, N-octyl ester with a peak area of 22.41% whereas the major compound in EMH was 5-(hydroxymethyl)-2-furancarboxaldehyde with a peak area of 44.32%. Most of the compounds in RMH are in the ester group, followed by alcohol while most compounds in EMH are ketones, followed by alcohol and esters.

## Discussion

This work aimed to compare the pH and phytochemical constituents between raw and methanol-extracted Kelulut, Tualang, and Manuka honeys. The slightly higher pH demonstrated in methanol-extracted honeys could be due to the elimination of some acidic compound during the process. The pH in raw Kelulut honey was consistent with the results reported previously in other studies [13, 14], which are also the same as raw Tualang honey [15-18] and Manuka honey [19]. The acidic property of honey is contributed by the presence of various organic acids, including amino, aromatic and aliphatic acids, which were also detected in this study.

There are marked differences between the components of raw and methanol-extracted honeys. Most of the volatile substances that are present in raw honey are diminished in the extracted form [20]. Certain bioactive components, but not all, become accentuated, for example; the benzene acetaldehyde, ketone aldehyde, and ketone furan group. Although the methanol-based extracts had been shown to have the most significant quantities of flavonoid, phenolic, terpenoid, and alkaloid substances [21-23], in this study we found that some phenols groups could be affected by the methanol extraction method as low peak areas were found in EKH compared to RKH and in ETH compared to RTH. This is also observed by Alevia et al. [24] and Ferreira et al. [25], whereby their results exhibited lower values for phenols, flavonoids, and antioxidant activity in methanolic extract honey.

This study showed that RKH, EKH, ETH, and EMH have ketone groups as their main bioactive components, with various forms, especially furan and pyran. The highest peak area in RKH and EKH is hydroxymethyl furfural, propanone, and protoanemonine, which were known to be associated with antimicrobial properties. RTH and ETH showed a high peak for the aldehyde compound, which is the hydroxymethyl-2-furancarboxaldehyde, followed by the ester group, which is the hexadecanoic acid. Hydroxymethyl furfural has recently been considered for the treatment of sickle cell disease [26], while hexadecanoic acid is known as an anti-inflammatory compound. RMH has ester groups as their main components which include fatty acids such as lauric, fumaric and palmitic acid, while the EMH is higher in hydroxymethyl-2-furancarboxaldehyde, furoic acid, and propanone. Fatty acids were demonstrated to have a broad spectrum of anti-microbiological activities against viruses and various bacteria [27]; while natural furan derivatives (e.g furoic acid) have been shown to have antimicrobial activity through selective inhibition of microbial growth and modification of enzymes [28].

The presence of various bioactive components in honey depends on many factors such as the pollen source, climate, environmental conditions, and the processing it undergoes [29,30]. In this study, both Kelulut and Tualang are local honeys, but not Manuka. Both Kelulut and Tualang bees live in tropical areas that have a variety of plants all year while the bees for Manuka honey live in a four-season climate with different kinds of plants. The differences between Kelulut and Tualang honey may be because Tualang wild bees forage and feed on a wider range of rare plant nectar in the forest than domestic Kelulut honey bees, which forage only a few metres from their hives.

The limitation of this study is that only one sample from each type of honey was used for analysis, and the preparation was made only for screening crude extracts of the phytochemical compound and not for specific bioactive compounds.

## Conclusions

The finding revealed that the phytochemical analysis of each raw honey and its methanol-extracted varieties showed differences in their most abundant bioactive compound group. In raw honey, the most abundant compound groups in RKH, RTH, and RMH are the ketone, alcohol, and ester groups, respectively. For methanol-extracted honey, all types of honey have ketone as their most abundant compound group. In addition, this study also showed that methanol-based honey extraction led to higher quantities of specific substances. However, the drawback is that some components such as phenols were reduced. Suitable extraction methods that minimise loss of important substances should be sought to obtain specific bioactive compounds that can be useful for pharmacological use.
